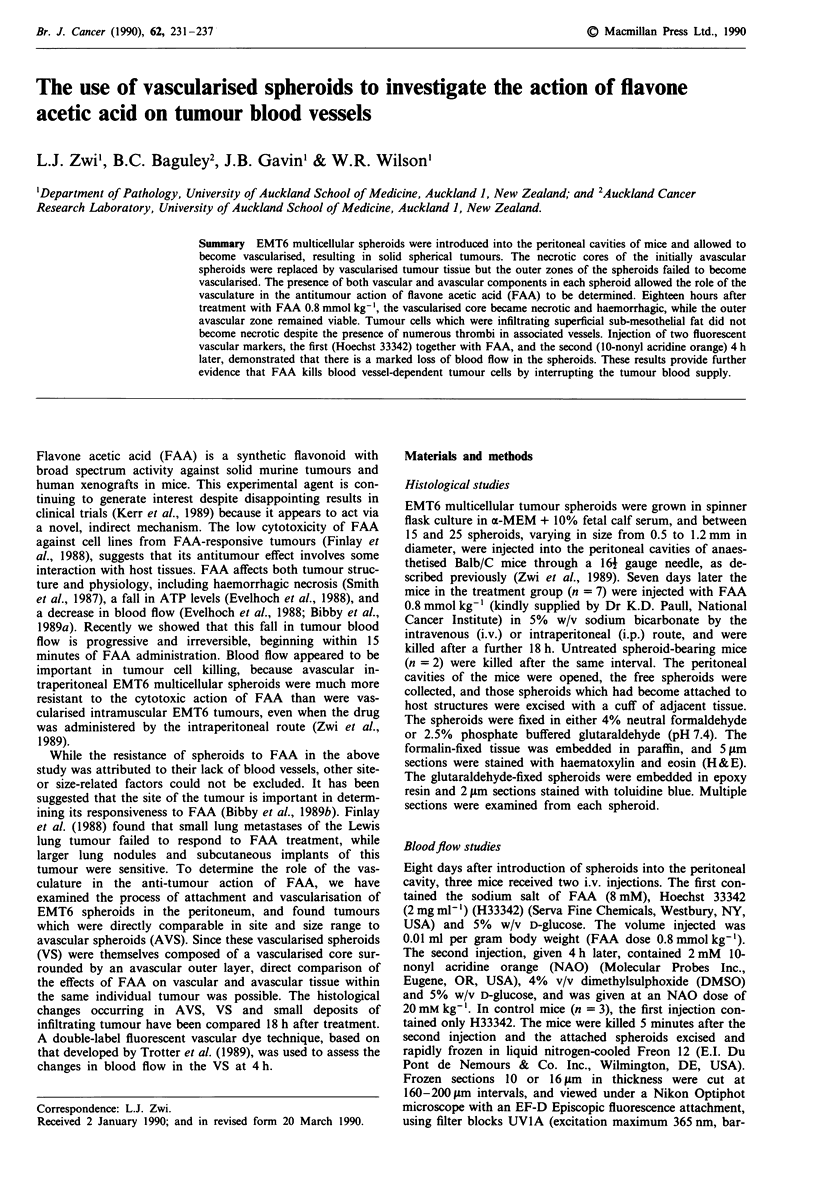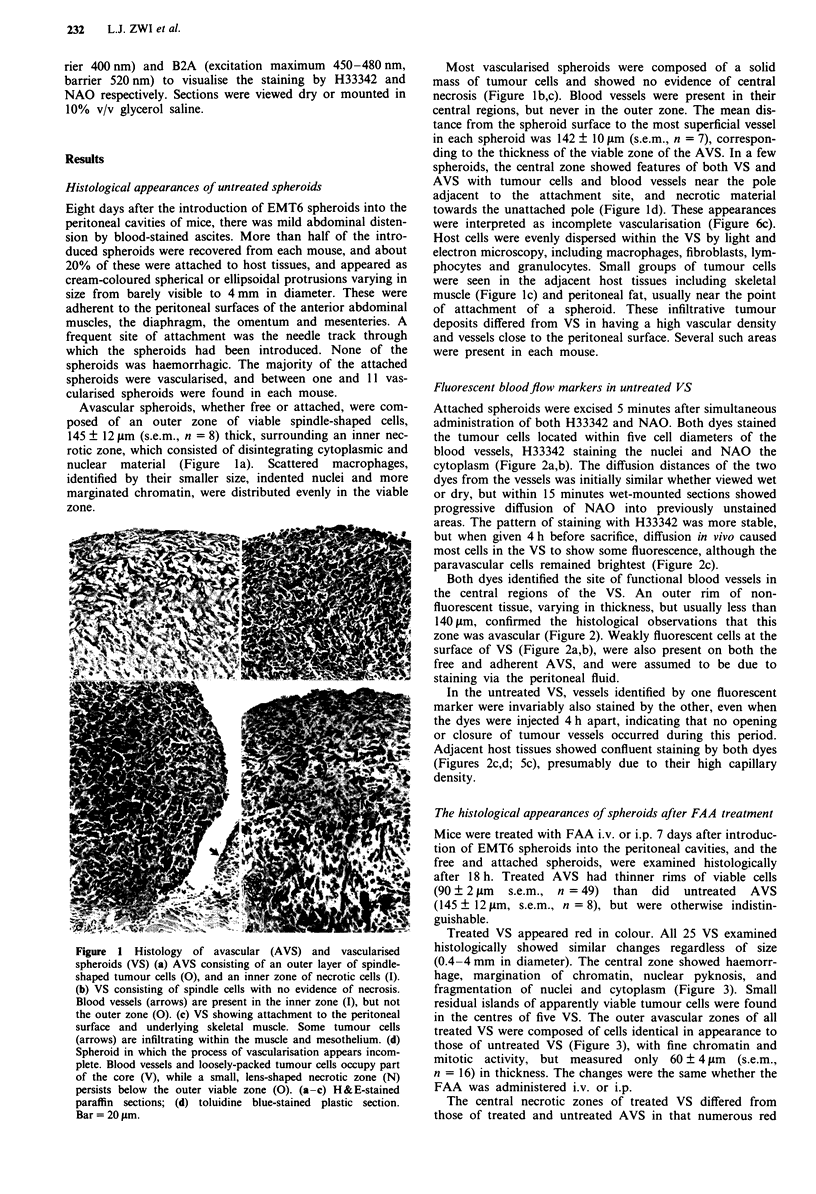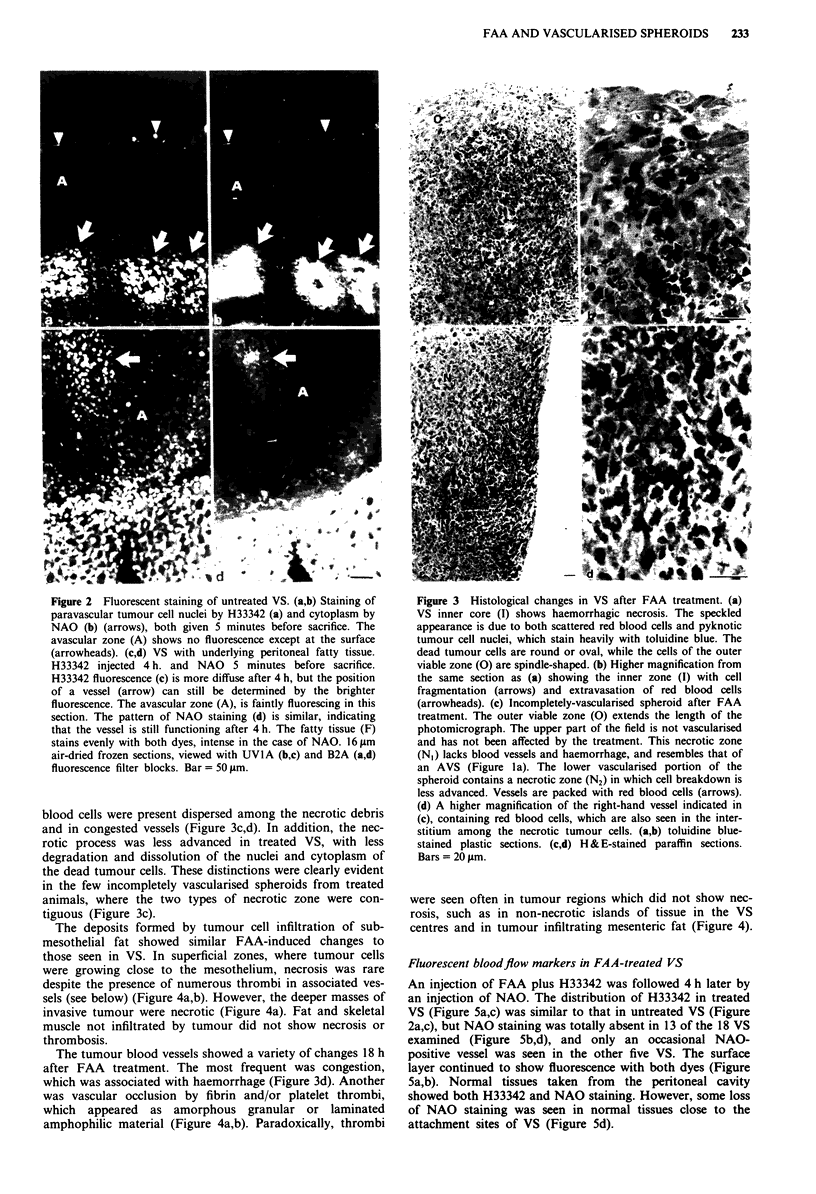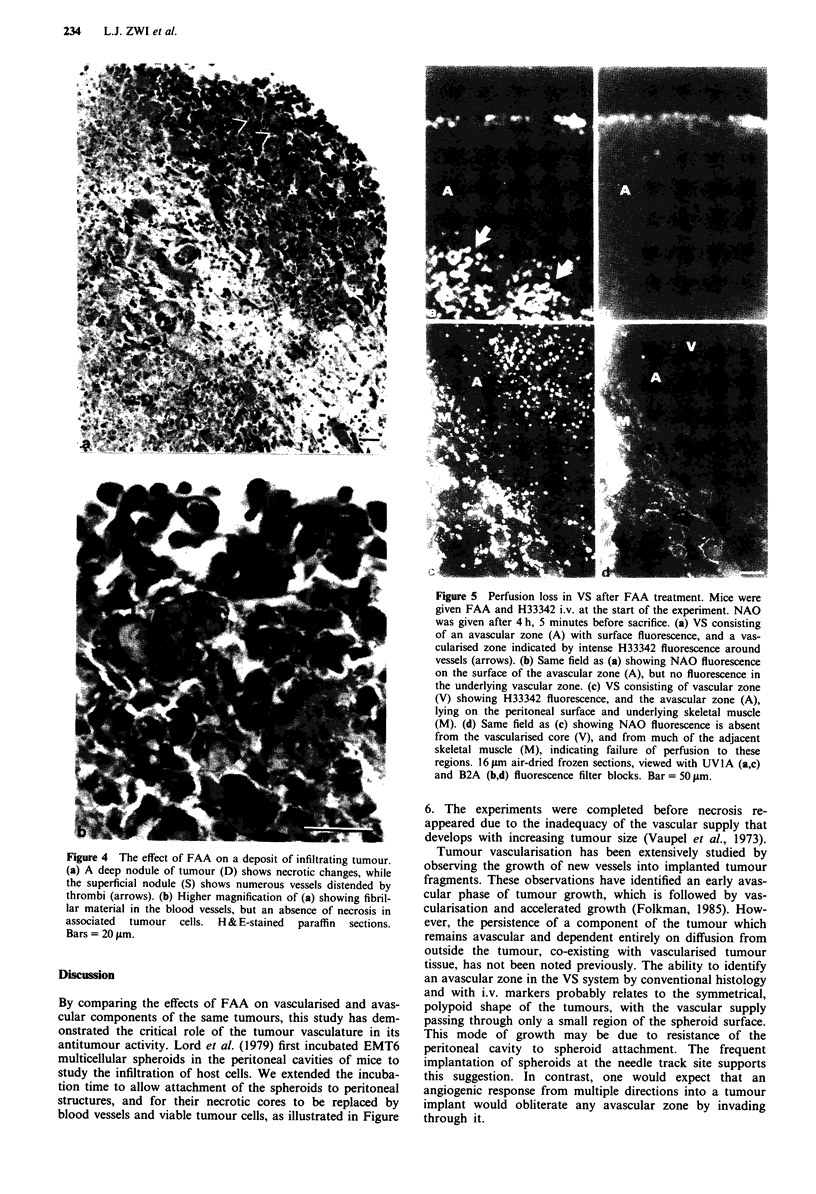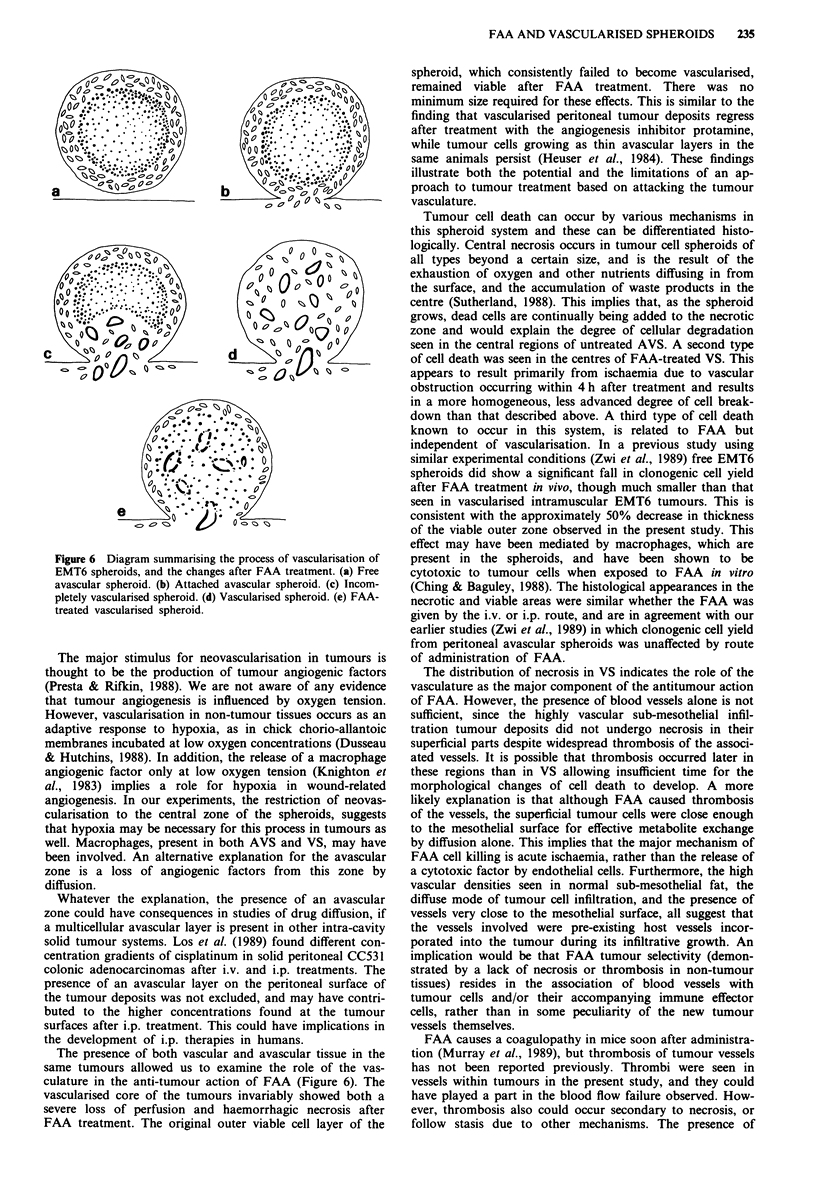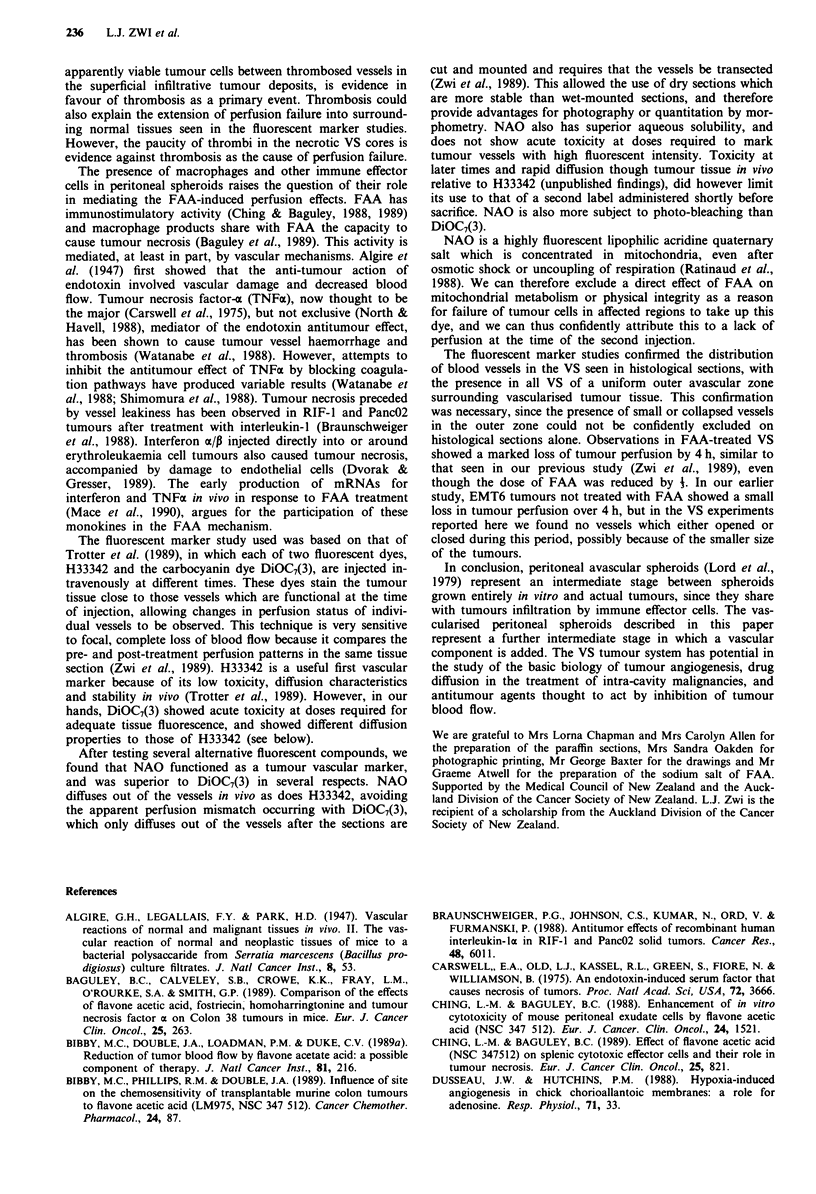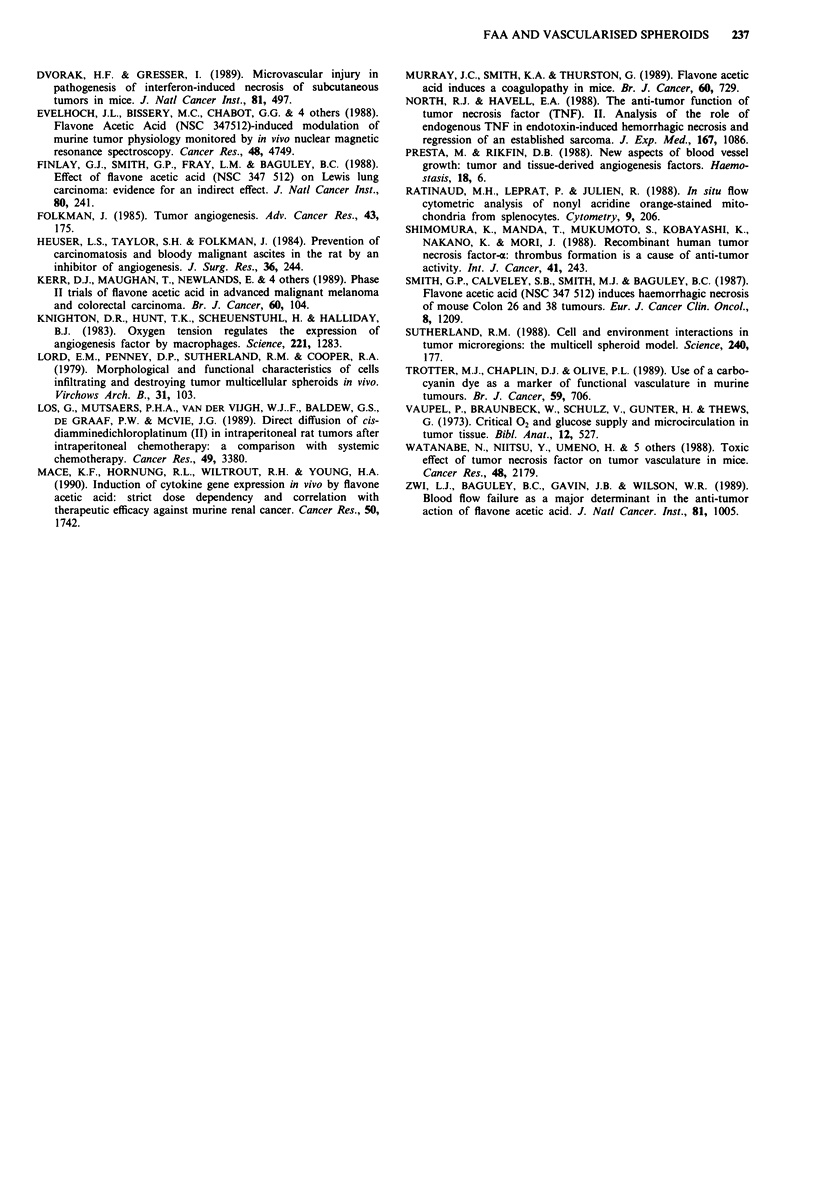# The use of vascularised spheroids to investigate the action of flavone acetic acid on tumour blood vessels.

**DOI:** 10.1038/bjc.1990.266

**Published:** 1990-08

**Authors:** L. J. Zwi, B. C. Baguley, J. B. Gavin, W. R. Wilson

**Affiliations:** Department of Pathology, University of Auckland School of Medicine, New Zealand.

## Abstract

**Images:**


Br. J. Cancer (1990), 62, 231-237 © Macmillan Press Ltd., 1990

### The use of vascularised spheroids to investigate the action of flavone acetic acid on tumour blood vessels

L.J. Zwi[1], B.C. Baguley[2], J.B. Gavin[1] & W.R. Wilson[1]

[1]*Department of Pathology, University of Auckland School of Medicine, Auckland 1, New Zealand; and* [2]*Auckland Cancer Research Laboratory, University of Auckland School of Medicine, Auckland 1, New Zealand.*

**Summary** EMT6 multicellular spheroids were introduced into the peritoneal cavities of mice and allowed to become vascularised, resulting in solid spherical tumours. The necrotic cores of the initially avascular spheroids were replaced by vascularised tumour tissue but the outer zones of the spheroids failed to become vascularised. The presence of both vascular and avascular components in each spheroid allowed the role of the vasculature in the antitumour action of flavone acetic acid (FAA) to be determined. Eighteen hours after treatment with FAA 0.8 mmol $kg^{-1}$, the vascularised core became necrotic and haemorrhagic, while the outer avascular zone remained viable. Tumour cells which were infiltrating superficial sub-mesothelial fat did not become necrotic despite the presence of numerous thrombi in associated vessels. Injection of two fluorescent vascular markers, the first (Hoechst 33342) together with FAA, and the second (10-nonyl acridine orange) 4 h later, demonstrated that there is a marked loss of blood flow in the spheroids. These results provide further evidence that FAA kills blood vessel-dependent tumour cells by interrupting the tumour blood supply.

Flavone acetic acid (FAA) is a synthetic flavonoid with broad spectrum activity against solid murine tumours and human xenografts in mice. This experimental agent is continuing to generate interest despite disappointing results in clinical trials (Kerr *et al.*, 1989) because it appears to act via a novel, indirect mechanism. The low cytotoxicity of FAA against cell lines from FAA-responsive tumours (Finlay *et al.*, 1988), suggests that its antitumour effect involves some interaction with host tissues. FAA affects both tumour structure and physiology, including haemorrhagic necrosis (Smith *et al.*, 1987), a fall in ATP levels (Evelhoch *et al.*, 1988), and a decrease in blood flow (Evelhoch *et al.*, 1988; Bibby *et al.*, 1989*a*). Recently we showed that this fall in tumour blood flow is progressive and irreversible, beginning within 15 minutes of FAA administration. Blood flow appeared to be important in tumour cell killing, because avascular intraperitoneal EMT6 multicellular spheroids were much more resistant to the cytotoxic action of FAA than were vascularised intramuscular EMT6 tumours, even when the drug was administered by the intraperitoneal route (Zwi *et al.*, 1989).

While the resistance of spheroids to FAA in the above study was attributed to their lack of blood vessels, other site- or size-related factors could not be excluded. It has been suggested that the site of the tumour is important in determining its responsiveness to FAA (Bibby *et al.*, 1989*b*). Finlay *et al.* (1988) found that small lung metastases of the Lewis lung tumour failed to respond to FAA treatment, while larger lung nodules and subcutaneous implants of this tumour were sensitive. To determine the role of the vasculature in the anti-tumour action of FAA, we have examined the process of attachment and vascularisation of EMT6 spheroids in the peritoneum, and found tumours which were directly comparable in site and size range to avascular spheroids (AVS). Since these vascularised spheroids (VS) were themselves composed of a vascularised core surrounded by an avascular outer layer, direct comparison of the effects of FAA on vascular and avascular tissue within the same individual tumour was possible. The histological changes occurring in AVS, VS and small deposits of infiltrating tumour have been compared 18 h after treatment. A double-label fluorescent vascular dye technique, based on that developed by Trotter *et al.* (1989), was used to assess the changes in blood flow in the VS at 4 h.

Correspondence: L.J. Zwi.
Received 2 January 1990; and in revised form 20 March 1990.

### Materials and methods

### *Histological studies*

EMT6 multicellular tumour spheroids were grown in spinner flask culture in α-MEM + 10% fetal calf serum, and between 15 and 25 spheroids, varying in size from 0.5 to 1.2 mm in diameter, were injected into the peritoneal cavities of anaesthetised Balb/C mice through a 16½ gauge needle, as described previously (Zwi *et al.*, 1989). Seven days later the mice in the treatment group ($n = 7$) were injected with FAA 0.8 mmol $kg^{-1}$ (kindly supplied by Dr K.D. Paull, National Cancer Institute) in 5% w/v sodium bicarbonate by the intravenous (i.v.) or intraperitoneal (i.p.) route, and were killed after a further 18 h. Untreated spheroid-bearing mice ($n = 2$) were killed after the same interval. The peritoneal cavities of the mice were opened, the free spheroids were collected, and those spheroids which had become attached to host structures were excised with a cuff of adjacent tissue. The spheroids were fixed in either 4% neutral formaldehyde or 2.5% phosphate buffered glutaraldehyde (pH 7.4). The formalin-fixed tissue was embedded in paraffin, and 5 μm sections were stained with haematoxylin and eosin (H&E). The glutaraldehyde-fixed spheroids were embedded in epoxy resin and 2 μm sections stained with toluidine blue. Multiple sections were examined from each spheroid.

### *Blood flow studies*

Eight days after introduction of spheroids into the peritoneal cavity, three mice received two i.v. injections. The first contained the sodium salt of FAA (8 mM), Hoechst 33342 (2 mg $ml^{-1}$) (H33342) (Serva Fine Chemicals, Westbury, NY, USA) and 5% w/v D-glucose. The volume injected was 0.01 ml per gram body weight (FAA dose 0.8 mmol $kg^{-1}$). The second injection, given 4 h later, contained 2 mM 10-nonyl acridine orange (NAO) (Molecular Probes Inc., Eugene, OR, USA), 4% v/v dimethylsulphoxide (DMSO) and 5% w/v D-glucose, and was given at an NAO dose of 20 mM $kg^{-1}$. In control mice ($n = 3$), the first injection contained only H33342. The mice were killed 5 minutes after the second injection and the attached spheroids excised and rapidly frozen in liquid nitrogen-cooled Freon 12 (E.I. Du Pont de Nemours & Co. Inc., Wilmington, DE, USA). Frozen sections 10 or 16 μm in thickness were cut at 160-200 μm intervals, and viewed under a Nikon Optiphot microscope with an EF-D Episcopic fluorescence attachment, using filter blocks UV1A (excitation maximum 365 nm, barrier 400 nm) and B2A (excitation maximum 450–480 nm, barrier 520 nm) to visualise the staining by H33342 and NAO respectively. Sections were viewed dry or mounted in 10% v/v glycerol saline.

### Results

### *Histological appearances of untreated spheroids*

Eight days after the introduction of EMT6 spheroids into the peritoneal cavities of mice, there was mild abdominal distension by blood-stained ascites. More than half of the introduced spheroids were recovered from each mouse, and about 20% of these were attached to host tissues, and appeared as cream-coloured spherical or ellipsoidal protrusions varying in size from barely visible to 4 mm in diameter. These were adherent to the peritoneal surfaces of the anterior abdominal muscles, the diaphragm, the omentum and mesenteries. A frequent site of attachment was the needle track through which the spheroids had been introduced. None of the spheroids was haemorrhagic. The majority of the attached spheroids were vascularised, and between one and 11 vascularised spheroids were found in each mouse.

Avascular spheroids, whether free or attached, were composed of an outer zone of viable spindle-shaped cells, $145 \pm 12\,\mu m$ (s.e.m., $n = 8$) thick, surrounding an inner necrotic zone, which consisted of disintegrating cytoplasmic and nuclear material (Figure 1a). Scattered macrophages, identified by their smaller size, indented nuclei and more marginated chromatin, were distributed evenly in the viable zone.

**Figure 1** Histology of avascular (AVS) and vascularised spheroids (VS) (**a**) AVS consisting of an outer layer of spindle-shaped tumour cells (O), and an inner zone of necrotic cells (I). (**b**) VS consisting of spindle cells with no evidence of necrosis. Blood vessels (arrows) are present in the inner zone (I), but not the outer zone (O). (**c**) VS showing attachment to the peritoneal surface and underlying skeletal muscle. Some tumour cells (arrows) are infiltrating within the muscle and mesothelium. (**d**) Spheroid in which the process of vascularisation appears incomplete. Blood vessels and loosely-packed tumour cells occupy part of the core (V), while a small, lens-shaped necrotic zone (N) persists below the outer viable zone (O). (**a–c**) H&E-stained paraffin sections; (**d**) toluidine blue-stained plastic section. Bar = 20 μm.

Most vascularised spheroids were composed of a solid mass of tumour cells and showed no evidence of central necrosis (Figure 1b,c). Blood vessels were present in their central regions, but never in the outer zone. The mean distance from the spheroid surface to the most superficial vessel in each spheroid was $142 \pm 10\,\mu m$ (s.e.m., $n = 7$), corresponding to the thickness of the viable zone of the AVS. In a few spheroids, the central zone showed features of both VS and AVS with tumour cells and blood vessels near the pole adjacent to the attachment site, and necrotic material towards the unattached pole (Figure 1d). These appearances were interpreted as incomplete vascularisation (Figure 6c). Host cells were evenly dispersed within the VS by light and electron microscopy, including macrophages, fibroblasts, lymphocytes and granulocytes. Small groups of tumour cells were seen in the adjacent host tissues including skeletal muscle (Figure 1c) and peritoneal fat, usually near the point of attachment of a spheroid. These infiltrative tumour deposits differed from VS in having a high vascular density and vessels close to the peritoneal surface. Several such areas were present in each mouse.

### *Fluorescent blood flow markers in untreated VS*

Attached spheroids were excised 5 minutes after simultaneous administration of both H33342 and NAO. Both dyes stained the tumour cells located within five cell diameters of the blood vessels, H33342 staining the nuclei and NAO the cytoplasm (Figure 2a,b). The diffusion distances of the two dyes from the vessels was initially similar whether viewed wet or dry, but within 15 minutes wet-mounted sections showed progressive diffusion of NAO into previously unstained areas. The pattern of staining with H33342 was more stable, but when given 4 h before sacrifice, diffusion *in vivo* caused most cells in the VS to show some fluorescence, although the paravascular cells remained brightest (Figure 2c).

Both dyes identified the site of functional blood vessels in the central regions of the VS. An outer rim of non-fluorescent tissue, varying in thickness, but usually less than 140 μm, confirmed the histological observations that this zone was avascular (Figure 2). Weakly fluorescent cells at the surface of VS (Figure 2a,b), were also present on both the free and adherent AVS, and were assumed to be due to staining via the peritoneal fluid.

In the untreated VS, vessels identified by one fluorescent marker were invariably also stained by the other, even when the dyes were injected 4 h apart, indicating that no opening or closure of tumour vessels occurred during this period. Adjacent host tissues showed confluent staining by both dyes (Figures 2c,d; 5c), presumably due to their high capillary density.

### *The histological appearances of spheroids after FAA treatment*

Mice were treated with FAA i.v. or i.p. 7 days after introduction of EMT6 spheroids into the peritoneal cavities, and the free and attached spheroids, were examined histologically after 18 h. Treated AVS had thinner rims of viable cells ($90 \pm 2\,\mu m$ s.e.m., $n = 49$) than did untreated AVS ($145 \pm 12\,\mu m$, s.e.m., $n = 8$), but were otherwise indistinguishable.

Treated VS appeared red in colour. All 25 VS examined histologically showed similar changes regardless of size (0.4–4 mm in diameter). The central zone showed haemorrhage, margination of chromatin, nuclear pyknosis, and fragmentation of nuclei and cytoplasm (Figure 3). Small residual islands of apparently viable tumour cells were found in the centres of five VS. The outer avascular zones of all treated VS were composed of cells identical in appearance to those of untreated VS (Figure 3), with fine chromatin and mitotic activity, but measured only $60 \pm 4\,\mu m$ (s.e.m., $n = 16$) in thickness. The changes were the same whether the FAA was administered i.v. or i.p.

The central necrotic zones of treated VS differed from those of treated and untreated AVS in that numerous red

**Figure 2** Fluorescent staining of untreated VS. (**a,b**) Staining of paravascular tumour cell nuclei by H33342 (**a**) and cytoplasm by NAO (**b**) (arrows), both given 5 minutes before sacrifice. The avascular zone (A) shows no fluorescence except at the surface (arrowheads). (**c,d**) VS with underlying peritoneal fatty tissue. H33342 injected 4 h. and NAO 5 minutes before sacrifice. H33342 fluorescence (**c**) is more diffuse after 4 h, but the position of a vessel (arrow) can still be determined by the brighter fluorescence. The avascular zone (A), is faintly fluorescing in this section. The pattern of NAO staining (**d**) is similar, indicating that the vessel is still functioning after 4 h. The fatty tissue (F) stains evenly with both dyes, intense in the case of NAO. 16 μm air-dried frozen sections, viewed with UV1A (**b,c**) and B2A (**a,d**) fluorescence filter blocks. Bar = 50 μm.

**Figure 3** Histological changes in VS after FAA treatment. (**a**) VS inner core (I) shows haemorrhagic necrosis. The speckled appearance is due to both scattered red blood cells and pyknotic tumour cell nuclei, which stain heavily with toluidine blue. The dead tumour cells are round or oval, while the cells of the outer viable zone (O) are spindle-shaped. (**b**) Higher magnification from the same section as (**a**) showing the inner zone (I) with cell fragmentation (arrows) and extravasation of red blood cells (arrowheads). (**c**) Incompletely-vascularised spheroid after FAA treatment. The outer viable zone (O) extends the length of the photomicrograph. The upper part of the field is not vascularised and has not been affected by the treatment. This necrotic zone ($N_1$) lacks blood vessels and haemorrhage, and resembles that of an AVS (Figure 1a). The lower vascularised portion of the spheroid contains a necrotic zone ($N_2$) in which cell breakdown is less advanced. Vessels are packed with red blood cells (arrows). (**d**) A higher magnification of the right-hand vessel indicated in (**c**), containing red blood cells, which are also seen in the interstitium among the necrotic tumour cells. (**a,b**) toluidine blue-stained plastic sections. (**c,d**) H&E-stained paraffin sections. Bars = 20 μm.

blood cells were present dispersed among the necrotic debris and in congested vessels (Figure 3c,d). In addition, the necrotic process was less advanced in treated VS, with less degradation and dissolution of the nuclei and cytoplasm of the dead tumour cells. These distinctions were clearly evident in the few incompletely vascularised spheroids from treated animals, where the two types of necrotic zone were contiguous (Figure 3c).

The deposits formed by tumour cell infiltration of submesothelial fat showed similar FAA-induced changes to those seen in VS. In superficial zones, where tumour cells were growing close to the mesothelium, necrosis was rare despite the presence of numerous thrombi in associated vessels (see below) (Figure 4a,b). However, the deeper masses of invasive tumour were necrotic (Figure 4a). Fat and skeletal muscle not infiltrated by tumour did not show necrosis or thrombosis.

The tumour blood vessels showed a variety of changes 18 h after FAA treatment. The most frequent was congestion, which was associated with haemorrhage (Figure 3d). Another was vascular occlusion by fibrin and/or platelet thrombi, which appeared as amorphous granular or laminated amphophilic material (Figure 4a,b). Paradoxically, thrombi were seen often in tumour regions which did not show necrosis, such as in non-necrotic islands of tissue in the VS centres and in tumour infiltrating mesenteric fat (Figure 4).

### *Fluorescent blood flow markers in FAA-treated VS*

An injection of FAA plus H33342 was followed 4 h later by an injection of NAO. The distribution of H33342 in treated VS (Figure 5a,c) was similar to that in untreated VS (Figure 2a,c), but NAO staining was totally absent in 13 of the 18 VS examined (Figure 5b,d), and only an occasional NAO-positive vessel was seen in the other five VS. The surface layer continued to show fluorescence with both dyes (Figure 5a,b). Normal tissues taken from the peritoneal cavity showed both H33342 and NAO staining. However, some loss of NAO staining was seen in normal tissues close to the attachment sites of VS (Figure 5d).

**Figure 4** The effect of FAA on a deposit of infiltrating tumour. (**a**) A deep nodule of tumour (D) shows necrotic changes, while the superficial nodule (S) shows numerous vessels distended by thrombi (arrows). (**b**) Higher magnification of (**a**) showing fibrillar material in the blood vessels, but an absence of necrosis in associated tumour cells. H&E-stained paraffin sections. Bars = 20 μm.

### Discussion

By comparing the effects of FAA on vascularised and avascular components of the same tumours, this study has demonstrated the critical role of the tumour vasculature in its antitumour activity. Lord *et al.* (1979) first incubated EMT6 multicellular spheroids in the peritoneal cavities of mice to study the infiltration of host cells. We extended the incubation time to allow attachment of the spheroids to peritoneal structures, and for their necrotic cores to be replaced by blood vessels and viable tumour cells, as illustrated in Figure

**Figure 5** Perfusion loss in VS after FAA treatment. Mice were given FAA and H33342 i.v. at the start of the experiment. NAO was given after 4 h, 5 minutes before sacrifice. (**a**) VS consisting of an avascular zone (A) with surface fluorescence, and a vascularised zone indicated by intense H33342 fluorescence around vessels (arrows). (**b**) Same field as (**a**) showing NAO fluorescence on the surface of the avascular zone (A), but no fluorescence in the underlying vascular zone. (**c**) VS consisting of vascular zone (V) showing H33342 fluorescence, and the avascular zone (A), lying on the peritoneal surface and underlying skeletal muscle (M). (**d**) Same field as (**c**) showing NAO fluorescence is absent from the vascularised core (V), and from much of the adjacent skeletal muscle (M), indicating failure of perfusion to these regions. 16 μm air-dried frozen sections, viewed with UV1A (**a**,**c**) and B2A (**b**,**d**) fluorescence filter blocks. Bar = 50 μm.

6. The experiments were completed before necrosis reappeared due to the inadequacy of the vascular supply that develops with increasing tumour size (Vaupel *et al.*, 1973).

Tumour vascularisation has been extensively studied by observing the growth of new vessels into implanted tumour fragments. These observations have identified an early avascular phase of tumour growth, which is followed by vascularisation and accelerated growth (Folkman, 1985). However, the persistence of a component of the tumour which remains avascular and dependent entirely on diffusion from outside the tumour, co-existing with vascularised tumour tissue, has not been noted previously. The ability to identify an avascular zone in the VS system by conventional histology and with i.v. markers probably relates to the symmetrical, polypoid shape of the tumours, with the vascular supply passing through only a small region of the spheroid surface. This mode of growth may be due to resistance of the peritoneal cavity to spheroid attachment. The frequent implantation of spheroids at the needle track site supports this suggestion. In contrast, one would expect that an angiogenic response from multiple directions into a tumour implant would obliterate any avascular zone by invading through it.

**Figure 6** Diagram summarising the process of vascularisation of EMT6 spheroids, and the changes after FAA treatment. (**a**) Free avascular spheroid. (**b**) Attached avascular spheroid. (**c**) Incompletely vascularised spheroid. (**d**) Vascularised spheroid. (**e**) FAA-treated vascularised spheroid.

The major stimulus for neovascularisation in tumours is thought to be the production of tumour angiogenic factors (Presta & Rifkin, 1988). We are not aware of any evidence that tumour angiogenesis is influenced by oxygen tension. However, vascularisation in non-tumour tissues occurs as an adaptive response to hypoxia, as in chick chorio-allantoic membranes incubated at low oxygen concentrations (Dusseau & Hutchins, 1988). In addition, the release of a macrophage angiogenic factor only at low oxygen tension (Knighton *et al.*, 1983) implies a role for hypoxia in wound-related angiogenesis. In our experiments, the restriction of neovascularisation to the central zone of the spheroids, suggests that hypoxia may be necessary for this process in tumours as well. Macrophages, present in both AVS and VS, may have been involved. An alternative explanation for the avascular zone is a loss of angiogenic factors from this zone by diffusion.

Whatever the explanation, the presence of an avascular zone could have consequences in studies of drug diffusion, if a multicellular avascular layer is present in other intra-cavity solid tumour systems. Los *et al.* (1989) found different concentration gradients of cisplatinum in solid peritoneal CC531 colonic adenocarcinomas after i.v. and i.p. treatments. The presence of an avascular layer on the peritoneal surface of the tumour deposits was not excluded, and may have contributed to the higher concentrations found at the tumour surfaces after i.p. treatment. This could have implications in the development of i.p. therapies in humans.

The presence of both vascular and avascular tissue in the same tumours allowed us to examine the role of the vasculature in the anti-tumour action of FAA (Figure 6). The vascularised core of the tumours invariably showed both a severe loss of perfusion and haemorrhagic necrosis after FAA treatment. The original outer viable cell layer of the spheroid, which consistently failed to become vascularised, remained viable after FAA treatment. There was no minimum size required for these effects. This is similar to the finding that vascularised peritoneal tumour deposits regress after treatment with the angiogenesis inhibitor protamine, while tumour cells growing as thin avascular layers in the same animals persist (Heuser *et al.*, 1984). These findings illustrate both the potential and the limitations of an approach to tumour treatment based on attacking the tumour vasculature.

Tumour cell death can occur by various mechanisms in this spheroid system and these can be differentiated histologically. Central necrosis occurs in tumour cell spheroids of all types beyond a certain size, and is the result of the exhaustion of oxygen and other nutrients diffusing in from the surface, and the accumulation of waste products in the centre (Sutherland, 1988). This implies that, as the spheroid grows, dead cells are continually being added to the necrotic zone and would explain the degree of cellular degradation seen in the central regions of untreated AVS. A second type of cell death was seen in the centres of FAA-treated VS. This appears to result primarily from ischaemia due to vascular obstruction occurring within 4 h after treatment and results in a more homogeneous, less advanced degree of cell breakdown than that described above. A third type of cell death known to occur in this system, is related to FAA but independent of vascularisation. In a previous study using similar experimental conditions (Zwi *et al.*, 1989) free EMT6 spheroids did show a significant fall in clonogenic cell yield after FAA treatment *in vivo*, though much smaller than that seen in vascularised intramuscular EMT6 tumours. This is consistent with the approximately 50% decrease in thickness of the viable outer zone observed in the present study. This effect may have been mediated by macrophages, which are present in the spheroids, and have been shown to be cytotoxic to tumour cells when exposed to FAA *in vitro* (Ching & Baguley, 1988). The histological appearances in the necrotic and viable areas were similar whether the FAA was given by the i.v. or i.p. route, and are in agreement with our earlier studies (Zwi *et al.*, 1989) in which clonogenic cell yield from peritoneal avascular spheroids was unaffected by route of administration of FAA.

The distribution of necrosis in VS indicates the role of the vasculature as the major component of the antitumour action of FAA. However, the presence of blood vessels alone is not sufficient, since the highly vascular sub-mesothelial infiltration tumour deposits did not undergo necrosis in their superficial parts despite widespread thrombosis of the associated vessels. It is possible that thrombosis occurred later in these regions than in VS allowing insufficient time for the morphological changes of cell death to develop. A more likely explanation is that although FAA caused thrombosis of the vessels, the superficial tumour cells were close enough to the mesothelial surface for effective metabolite exchange by diffusion alone. This implies that the major mechanism of FAA cell killing is acute ischaemia, rather than the release of a cytotoxic factor by endothelial cells. Furthermore, the high vascular densities seen in normal sub-mesothelial fat, the diffuse mode of tumour cell infiltration, and the presence of vessels very close to the mesothelial surface, all suggest that the vessels involved were pre-existing host vessels incorporated into the tumour during its infiltrative growth. An implication would be that FAA tumour selectivity (demonstrated by a lack of necrosis or thrombosis in non-tumour tissues) resides in the association of blood vessels with tumour cells and/or their accompanying immune effector cells, rather than in some peculiarity of the new tumour vessels themselves.

FAA causes a coagulopathy in mice soon after administration (Murray *et al.*, 1989), but thrombosis of tumour vessels has not been reported previously. Thrombi were seen in vessels within tumours in the present study, and they could have played a part in the blood flow failure observed. However, thrombosis also could occur secondary to necrosis, or follow stasis due to other mechanisms. The presence of apparently viable tumour cells between thrombosed vessels in the superficial infiltrative tumour deposits, is evidence in favour of thrombosis as a primary event. Thrombosis could also explain the extension of perfusion failure into surrounding normal tissues seen in the fluorescent marker studies. However, the paucity of thrombi in the necrotic VS cores is evidence against thrombosis as the cause of perfusion failure.

The presence of macrophages and other immune effector cells in peritoneal spheroids raises the question of their role in mediating the FAA-induced perfusion effects. FAA has immunostimulatory activity (Ching & Baguley, 1988, 1989) and macrophage products share with FAA the capacity to cause tumour necrosis (Baguley *et al.*, 1989). This activity is mediated, at least in part, by vascular mechanisms. Algire *et al.* (1947) first showed that the anti-tumour action of endotoxin involved vascular damage and decreased blood flow. Tumour necrosis factor-$\alpha$ (TNF$\alpha$), now thought to be the major (Carswell *et al.*, 1975), but not exclusive (North & Havell, 1988), mediator of the endotoxin antitumour effect, has been shown to cause tumour vessel haemorrhage and thrombosis (Watanabe *et al.*, 1988). However, attempts to inhibit the antitumour effect of TNF$\alpha$ by blocking coagulation pathways have produced variable results (Watanabe *et al.*, 1988; Shimomura *et al.*, 1988). Tumour necrosis preceded by vessel leakiness has been observed in RIF-1 and Panc02 tumours after treatment with interleukin-1 (Braunschweiger *et al.*, 1988). Interferon $\alpha/\beta$ injected directly into or around erythroleukaemia cell tumours also caused tumour necrosis, accompanied by damage to endothelial cells (Dvorak & Gresser, 1989). The early production of mRNAs for interferon and TNF$\alpha$ *in vivo* in response to FAA treatment (Mace *et al.*, 1990), argues for the participation of these monokines in the FAA mechanism.

The fluorescent marker study used was based on that of Trotter *et al.* (1989), in which each of two fluorescent dyes, H33342 and the carbocyanin dye $DiOC_7(3)$, are injected intravenously at different times. These dyes stain the tumour tissue close to those vessels which are functional at the time of injection, allowing changes in perfusion status of individual vessels to be observed. This technique is very sensitive to focal, complete loss of blood flow because it compares the pre- and post-treatment perfusion patterns in the same tissue section (Zwi *et al.*, 1989). H33342 is a useful first vascular marker because of its low toxicity, diffusion characteristics and stability *in vivo* (Trotter *et al.*, 1989). However, in our hands, $DiOC_7(3)$ showed acute toxicity at doses required for adequate tissue fluorescence, and showed different diffusion properties to those of H33342 (see below).

After testing several alternative fluorescent compounds, we found that NAO functioned as a tumour vascular marker, and was superior to $DiOC_7(3)$ in several respects. NAO diffuses out of the vessels *in vivo* as does H33342, avoiding the apparent perfusion mismatch occurring with $DiOC_7(3)$, which only diffuses out of the vessels after the sections are cut and mounted and requires that the vessels be transected (Zwi *et al.*, 1989). This allowed the use of dry sections which are more stable than wet-mounted sections, and therefore provide advantages for photography or quantitation by morphometry. NAO also has superior aqueous solubility, and does not show acute toxicity at doses required to mark tumour vessels with high fluorescent intensity. Toxicity at later times and rapid diffusion though tumour tissue *in vivo* relative to H33342 (unpublished findings), did however limit its use to that of a second label administered shortly before sacrifice. NAO is also more subject to photo-bleaching than $DiOC_7(3)$.

NAO is a highly fluorescent lipophilic acridine quaternary salt which is concentrated in mitochondria, even after osmotic shock or uncoupling of respiration (Ratinaud *et al.*, 1988). We can therefore exclude a direct effect of FAA on mitochondrial metabolism or physical integrity as a reason for failure of tumour cells in affected regions to take up this dye, and we can thus confidently attribute this to a lack of perfusion at the time of the second injection.

The fluorescent marker studies confirmed the distribution of blood vessels in the VS seen in histological sections, with the presence in all VS of a uniform outer avascular zone surrounding vascularised tumour tissue. This confirmation was necessary, since the presence of small or collapsed vessels in the outer zone could not be confidently excluded on histological sections alone. Observations in FAA-treated VS showed a marked loss of tumour perfusion by 4 h, similar to that seen in our previous study (Zwi *et al.*, 1989), even though the dose of FAA was reduced by ⅓. In our earlier study, EMT6 tumours not treated with FAA showed a small loss in tumour perfusion over 4 h, but in the VS experiments reported here we found no vessels which either opened or closed during this period, possibly because of the smaller size of the tumours.

In conclusion, peritoneal avascular spheroids (Lord *et al.*, 1979) represent an intermediate stage between spheroids grown entirely *in vitro* and actual tumours, since they share with tumours infiltration by immune effector cells. The vascularised peritoneal spheroids described in this paper represent a further intermediate stage in which a vascular component is added. The VS tumour system has potential in the study of the basic biology of tumour angiogenesis, drug diffusion in the treatment of intra-cavity malignancies, and antitumour agents thought to act by inhibition of tumour blood flow.

We are grateful to Mrs Lorna Chapman and Mrs Carolyn Allen for the preparation of the paraffin sections, Mrs Sandra Oakden for photographic printing, Mr George Baxter for the drawings and Mr Graeme Atwell for the preparation of the sodium salt of FAA. Supported by the Medical Council of New Zealand and the Auckland Division of the Cancer Society of New Zealand. L.J. Zwi is the recipient of a scholarship from the Auckland Division of the Cancer Society of New Zealand.

### References

ALGIRE, G.H., LEGALLAIS, F.Y. & PARK, H.D. (1947). Vascular reactions of normal and malignant tissues *in vivo*. II. The vascular reaction of normal and neoplastic tissues of mice to a bacterial polysaccharide from *Serratia marcescens* (*Bacillus prodigiosus*) culture filtrates. *J. Natl Cancer Inst.*, **8**, 53.

BAGULEY, B.C., CALVELEY, S.B., CROWE, K.K., FRAY, L.M., O'ROURKE, S.A. & SMITH, G.P. (1989). Comparison of the effects of flavone acetic acid, fostriecin, homoharringtonine and tumour necrosis factor $\alpha$ on Colon 38 tumours in mice. *Eur. J. Cancer Clin. Oncol.*, **25**, 263.

BIBBY, M.C., DOUBLE, J.A., LOADMAN, P.M. & DUKE, C.V. (1989*a*). Reduction of tumor blood flow by flavone acetate acid: a possible component of therapy. *J. Natl Cancer Inst.*, **81**, 216.

BIBBY, M.C., PHILLIPS, R.M. & DOUBLE, J.A. (1989). Influence of site on the chemosensitivity of transplantable murine colon tumours to flavone acetic acid (LM975, NSC 347 512). *Cancer Chemother. Pharmacol.*, **24**, 87.

BRAUNSCHWEIGER, P.G., JOHNSON, C.S., KUMAR, N., ORD, V. & FURMANSKI, P. (1988). Antitumor effects of recombinant human interleukin-1$\alpha$ in RIF-1 and Panc02 solid tumors. *Cancer Res.*, **48**, 6011.

CARSWELL,, E.A., OLD, L.J., KASSEL, R.L., GREEN, S., FIORE, N. & WILLIAMSON, B. (1975). An endotoxin-induced serum factor that causes necrosis of tumors. *Proc. Natl Acad. Sci, USA*, **72**, 3666.

CHING, L.-M. & BAGULEY, B.C. (1988). Enhancement of *in vitro* cytotoxicity of mouse peritoneal exudate cells by flavone acetic acid (NSC 347 512). *Eur. J. Cancer. Clin. Oncol.*, **24**, 1521.

CHING, L.-M. & BAGULEY, B.C. (1989). Effect of flavone acetic acid (NSC 347512) on splenic cytotoxic effector cells and their role in tumour necrosis. *Eur. J. Cancer Clin. Oncol.*, **25**, 821.

DUSSEAU, J.W. & HUTCHINS, P.M. (1988). Hypoxia-induced angiogenesis in chick chorioallantoic membranes: a role for adenosine. *Resp. Physiol.*, **71**, 33.

DVORAK, H.F. & GRESSER, I. (1989). Microvascular injury in pathogenesis of interferon-induced necrosis of subcutaneous tumors in mice. *J. Natl Cancer Inst.*, **81**, 497.

EVELHOCH, J.L., BISSERY, M.C., CHABOT, G.G. & 4 others (1988). Flavone Acetic Acid (NSC 347512)-induced modulation of murine tumor physiology monitored by *in vivo* nuclear magnetic resonance spectroscopy. *Cancer Res.*, **48**, 4749.

FINLAY, G.J., SMITH, G.P., FRAY, L.M. & BAGULEY, B.C. (1988). Effect of flavone acetic acid (NSC 347 512) on Lewis lung carcinoma: evidence for an indirect effect. *J. Natl Cancer Inst.*, **80**, 241.

FOLKMAN, J. (1985). Tumor angiogenesis. *Adv. Cancer Res.*, **43**, 175.

HEUSER, L.S., TAYLOR, S.H. & FOLKMAN, J. (1984). Prevention of carcinomatosis and bloody malignant ascites in the rat by an inhibitor of angiogenesis. *J. Surg. Res.*, **36**, 244.

KERR, D.J., MAUGHAN, T., NEWLANDS, E. & 4 others (1989). Phase II trials of flavone acetic acid in advanced malignant melanoma and colorectal carcinoma. *Br. J. Cancer*, **60**, 104.

KNIGHTON, D.R., HUNT, T.K., SCHEUENSTUHL, H. & HALLIDAY, B.J. (1983). Oxygen tension regulates the expression of angiogenesis factor by macrophages. *Science*, **221**, 1283.

LORD, E.M., PENNEY, D.P., SUTHERLAND, R.M. & COOPER, R.A. (1979). Morphological and functional characteristics of cells infiltrating and destroying tumor multicellular spheroids *in vivo*. *Virchows Arch. B.*, **31**, 103.

LOS, G., MUTSAERS, P.H.A., VAN DER VIJGH, W.J..F., BALDEW, G.S., DE GRAAF, P.W. & MCVIE, J.G. (1989). Direct diffusion of *cis*-diamminedichloroplatinum (II) in intraperitoneal rat tumors after intraperitoneal chemotherapy: a comparison with systemic chemotherapy. *Cancer Res.*, **49**, 3380.

MACE, K.F., HORNUNG, R.L., WILTROUT, R.H. & YOUNG, H.A. (1990). Induction of cytokine gene expression *in vivo* by flavone acetic acid: strict dose dependency and correlation with therapeutic efficacy against murine renal cancer. *Cancer Res.*, **50**, 1742.

MURRAY, J.C., SMITH, K.A. & THURSTON, G. (1989). Flavone acetic acid induces a coagulopathy in mice. *Br. J. Cancer*, **60**, 729.

NORTH, R.J. & HAVELL, E.A. (1988). The anti-tumor function of tumor necrosis factor (TNF). II. Analysis of the role of endogenous TNF in endotoxin-induced hemorrhagic necrosis and regression of an established sarcoma. *J. Exp. Med.*, **167**, 1086.

PRESTA, M. & RIKFIN, D.B. (1988). New aspects of blood vessel growth: tumor and tissue-derived angiogenesis factors. *Haemostasis*, **18**, 6.

RATINAUD, M.H., LEPRAT, P. & JULIEN, R. (1988). *In situ* flow cytometric analysis of nonyl acridine orange-stained mitochondria from splenocytes. *Cytometry*, **9**, 206.

SHIMOMURA, K., MANDA, T., MUKUMOTO, S., KOBAYASHI, K., NAKANO, K. & MORI, J. (1988). Recombinant human tumor necrosis factor-α: thrombus formation is a cause of anti-tumor activity. *Int. J. Cancer*, **41**, 243.

SMITH, G.P., CALVELEY, S.B., SMITH, M.J. & BAGULEY, B.C. (1987). Flavone acetic acid (NSC 347 512) induces haemorrhagic necrosis of mouse Colon 26 and 38 tumours. *Eur. J. Cancer Clin. Oncol.*, **8**, 1209.

SUTHERLAND, R.M. (1988). Cell and environment interactions in tumor microregions: the multicell spheroid model. *Science*, **240**, 177.

TROTTER, M.J., CHAPLIN, D.J. & OLIVE, P.L. (1989). Use of a carbocyanin dye as a marker of functional vasculature in murine tumours. *Br. J. Cancer*, **59**, 706.

VAUPEL, P., BRAUNBECK, W., SCHULZ, V., GUNTER, H. & THEWS, G. (1973). Critical $O_2$ and glucose supply and microcirculation in tumor tissue. *Bibl. Anat.*, **12**, 527.

WATANABE, N., NIITSU, Y., UMENO, H. & 5 others (1988). Toxic effect of tumor necrosis factor on tumor vasculature in mice. *Cancer Res.*, **48**, 2179.

ZWI, L.J., BAGULEY, B.C., GAVIN, J.B. & WILSON, W.R. (1989). Blood flow failure as a major determinant in the anti-tumor action of flavone acetic acid. *J. Natl Cancer. Inst.*, **81**, 1005.